# Time-band network model and binary tree algorithm for multimodal irregular flight recovery

**DOI:** 10.1038/s41598-024-56000-w

**Published:** 2024-03-04

**Authors:** Peinan He

**Affiliations:** College of Air Traffic Management, Civil Aviation Flight University of China, Guanghan, 618307 China

**Keywords:** Recovery of irregular flights, Multimodal time-band network, Binary tree generation algorithm, Engineering, Mathematics and computing

## Abstract

Recovery of irregular flights caused by various reasons such as aircraft failures and airport closures is studied in this research and a multimodal time-band network model for solving the issue is proposed. It transforms the flight routing problem into a time-based network, which is used to obtain the delay and cancellation costs of each flight. With delay and cancellation costs as variables, the proposed model aims to minimize recovery costs under constraints. This research also suggests a developed binary tree algorithm, which improves the efficiency of model solving. The results show that the rescheduled flights and re-selected flight routes are at the lowest cost and helpful to achieve a balance of flight flow without affecting flight safety. This method used in this work shows its certain value in helping airlines restore flight operations in the shortest possible time and at the lowest cost, improving operational efficiency and service quality.

## Introduction

Irregular flights not only cause huge economic losses to airlines but also affect the travel experience of aviation consumers. To assist airlines in redefining flight plans in the shortest possible time and at the lowest cost, reconstructing aircraft routes, and restoring flight operations, the study of optimization methods for restoring irregular flights has important significance and application value.

Various models and algorithms have been proposed to solve the problem of restoring irregular flights. A spatiotemporal network optimization model was proposed for reconstructing delayed flight routes^1^. A comprehensive fundamental model for flight recovery^2^ and an optimization model^3^ were reported to provide solutions for different flight interruption scenarios. The greedy random fitness search algorithm was introduced for re-planning flights that had been waiting and delayed on the ground^4^. A quantitative mechanism has been developed to evaluate the degree of impact on flights with operational interruptions and provide actionable recovery recommendations^5^. A method integrating flight planning models and resource allocation optimization models has been proposed to minimize flight delays and cancellations^6^. The column generation algorithm^7^ is introduced to solve irregular flight recovery model. Greedy algorithms and random algorithms^8^ can be used to modify daily flight schedules to minimize the overall operational costs of airlines. The particle swarm algorithm^9^ and a dynamic decision algorithm^10^ have also been mentioned for providing model-solving solutions. The details of the effective transit time prediction model for flights can be found in the reference^11^. Recently, a data-driven heuristic method^12^ and the Floyd Warhill algorithm^13^ were also invoked to solve the problem of irregular flight recovery under specific conditions.

It is difficult for airports to avoid unpredictable events in their daily operations. For example, sudden extreme weather, aircraft malfunctions, or airport closures due to unforeseen circumstances. From the literature mentioned above, it can be seen that irregular flight recovery problems caused by different reasons have different impacts and design preferences for applicable solutions. This article proposes a multimodal spatiotemporal network model for irregular flight recovery, taking into account the operational quality of airlines, in response to unpredictable events at airports. The core is to transform the path problem into a time-based network, and then use this network to evaluate the delay and cancellation costs of each flight, with delay and cancellation costs as variables, to achieve the lowest recovery cost under constraint conditions. Meanwhile, this article proposes a binary tree generation algorithm to search for the best solution with high efficiency.

The main content of this article includes constructing a spatiotemporal network model for multimodal irregular flight recovery, establishing a mathematical model for restoring irregular flights and setting up its constraints, developing a binary tree generation algorithm, and designing and programming it by using C++ to evaluate and solve the model; finding the costs of restoring irregular flights, and presenting rescheduled flights and re-selected routes.

## Multimodal time-band network model

Multimodal theory and technology have been widely applied in various fields to solve practical problems, such as the multimodal classification method for road infrastructure distress images, and verify the effectiveness of the proposed method for various types of distress using distress images obtained from reality^14^. By utilizing the multimodal information of eye movements and combining it with brain–computer interfaces based on electroencephalography, a new object detection system has been formed, further improving the performance of the object detection system^15^. Using multimodal information generated by mirror defect refraction for automatic defect detection of mirrors, combined with deep neural networks to obtain input images for deflection surgery, can help improve manufacturing processes and quality^16^. The self-supervised deep completion method based on multimodal spatiotemporal consistency is considered superior to other self-supervised networks and even surpasses some supervised networks^17^. The hierarchical estimation method using multimodal state programming was applied to study the regression problem of small and large difficulty datasets, and the results improved by 232% and 62%, respectively^18^. Using multimodal dynamic programming algorithms, different transmission networks are mapped, and then various graphs are pruned using preprocessing techniques. It is believed that this method can almost immediately provide users with efficient multimodal solutions^19^, and so on.

According to the concept of multimodality, for each flight, whether it is a normal or an irregular flight caused by various reasons, there are several modes of aircraft in operation. For example, takeoff, cruise, and landing, as well as various milestone events such as delays, re-routings, and cancellations, are collectively referred to as multimodal events. Every flight, regardless of its modes, has spatiotemporal characteristics and a series of precise spatiotemporal data to describe its status. These multi-modal spatiotemporal data fully describe the holographic feature information related to the task object, with completeness, orthogonality, correlation, and intuitiveness. Therefore, this article uses multimodal fusion technology to study a time network model for restoring irregular flights, which demonstrates a certain novelty.

### Time-band network description of the sample

This article takes Sichuan Airlines as an example to conduct research. By the end of 2022, the company had 184 Airbus A350-A320 series aircraft, ranking fifth in the country in terms of aircraft quantity and also the largest Airbus fleet in China. Sichuan Airlines has a total of 1415 domestic flights nationwide every day, involving 99 airports. We collected some operation data for research, involving four airports in Beijing, Chengdu, Kunming, and Nanchang. There were three aircraft in operation assigned to 12 flights involved, with each of them operating 4 flights, as shown in Table 1.

**Table 1:** Original flight schedule (total cost = $583,274).

Aircraft no	Flight ID	Origin	Destination	Departure	Arrival	Duration	Cancellation cost
Aircraft 1	3U11	PEK	CTU	08:00	11:10	3:10	72,053
	3U12	CTU	KMG	11:50	13:30	1:40	42,168
	3U13	KMG	CTU	14:10	15:40	1:30	46,262
	3U14	CTU	PEK	16:20	19:00	2:40	61,000
Aircraft 2	3U21	CTU	PEK	11:20	14:00	2:40	61,000
	3U22	PEK	CTU	14:40	17:50	3:10	72,053
	3U23	CTU	KMG	18:30	20:10	1:40	42,168
	3U24	KMG	CTU	20:50	22:20	1:30	46,262
Aircraft 3	3U31	KMG	KNH	10:55	13:10	2:15	38,430
	3U32	KNH	KMG	13:50	16:20	2:30	31,724
	3U33	KMG	KNH	17:00	19:15	2:15	38,430
	3U34	KNH	KMG	19:55	22:25	2:30	31,724
Total							583,274

**Figure 1:**
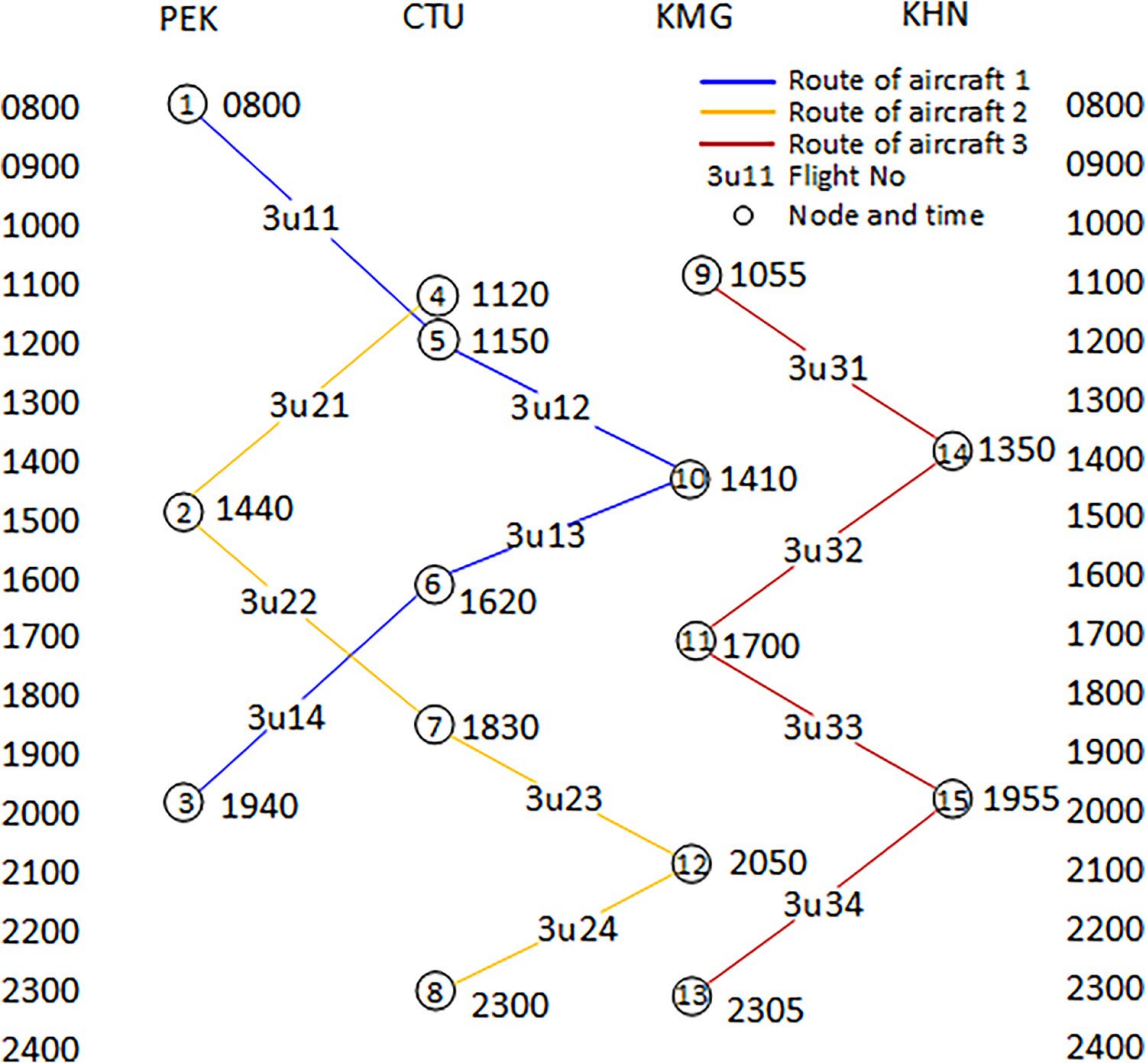
Time-band representation network of the sample.

According to the aircraft and flight data in Table 1, a time-band network structure diagram of irregular flights is constructed, as shown in Fig. 1. This time-band network is composed of flight nodes, flight schedules, and aircraft routes. The horizontal axis represents space, which is the airport where the flight is located. The vertical axis represents the departure and arrival time of flights, with a time sequence from top to bottom until the final destination of the last flight (Station-sink nodes).

In Fig. 1, the time interval is 30 min, and the shortest connecting time of the flights is set to 40 min. The flight schedule in the figure is shown in hours plus minutes. For example, 0800 indicates 08:00. The three routes in the figure correspond to the flights of the three aircraft in Table 1. The generation method of the time-band network is as follows.

From Table 1, it can be seen that at the initial moment, Aircraft 1 has parked at Beijing Airport. Based on the departure time of flight 3U11, it can be established that the earliest available time for Aircraft 1 is 08:00. Therefore, taking the available time and airport into account for Aircraft 1, Node 1 can be settled in the network diagram. Set the marking time of this node to 0800, indicating that Node 1 has aircraft in operation at 08:00. Subsequently, Aircraft 1 is taking off from Node 1 and operating flight 3U11. After flying for 190 min, it lands at Chengdu Shuangliu Airport at 11:10 (Node 5). At this point, the aircraft needs to meet the minimum connecting time of 40 min. The available time for Aircraft 1 at Chengdu Shuangliu Airport is 11:50. And then its next task is 3U12, with a node time of 11:50. It will arrive at Kunming Airport after a flight of 100 min. The flight followed is 3U13, with a node time of 14:10. It will arrive at Chengdu Shuangliu Airport after flying for 90 min. Finally, aircraft 1 operating 3U14 takes off from Chengdu at 16:20 and lands in Beijing after flying for 160 min, at 19:00. At that moment, aircraft 1 has completed all flight tasks for the day and is stationed at Beijing Capital Airport.

The first airport for Aircraft 2 is Chengdu Shuangliu Airport, operating flight 3U21. It will depart from Node 4 at 11:20 and land in Beijing at 14:00 after flying 160 min. Meeting the minimum connecting time of 40 min, flight 3U22 will take off at 14:40 and land at Chengdu Shuangliu Airport at 17:50. In 40 min, flight 3U23 will depart at 18:30 and land at Kunming Airport at 20:10. Meeting the minimum connecting time of 40 min, flight 3U22 is taking off at 14:40 and landing at Chengdu Airport at 17:50. 40 min later, flight 3U23 is taking off at 18:30 and landing at Kunming Airport at 20:10. 40 min later, flight 3U24 will take off at 20:50. After flying for 90 min, it lands at the destination airport Chengdu Shuangliu at 22:20, completing all flight tasks for the day and staying in Chengdu.

Similarly, the first airport for Aircraft 3 is Kunming Airport. Flight 3U31 will depart at 10:55 and will land at Nanchang Airport at 13:10 after flying 135 min. In 40 min, flight 3U32 will take off at 13:50 and land at Kunming Airport at 16:20. After 40 min, flight 3U33 will depart at 17:00 and land at Kunming Airport at 19:15. After that, in 40 min, flight 3U34 will depart at 19:55 and return to Kunming around 22:25, then stay overnight.

In this way, a complete time-band network diagram of normal flights is obtained. Meanwhile, it can be found that the network is not constructed based on distance, but rather on time. Therefore, it is called a time-band network diagram.

### Time-band network structures of multimodal irregular flight recovery

The reasons for irregular flights are various, such as aircraft failure and airport closure. The normal flight time-band network construction method is used to construct time-band network diagrams for irregular flight recovery in irregular scenarios. The case settings are as follows:

- *Scenario 1* Aircraft failure: Assumed that aircraft 2 has malfunctioned at 10:00 and cannot be repaired before 23:00, it is cancelled.

**Figure 2:**
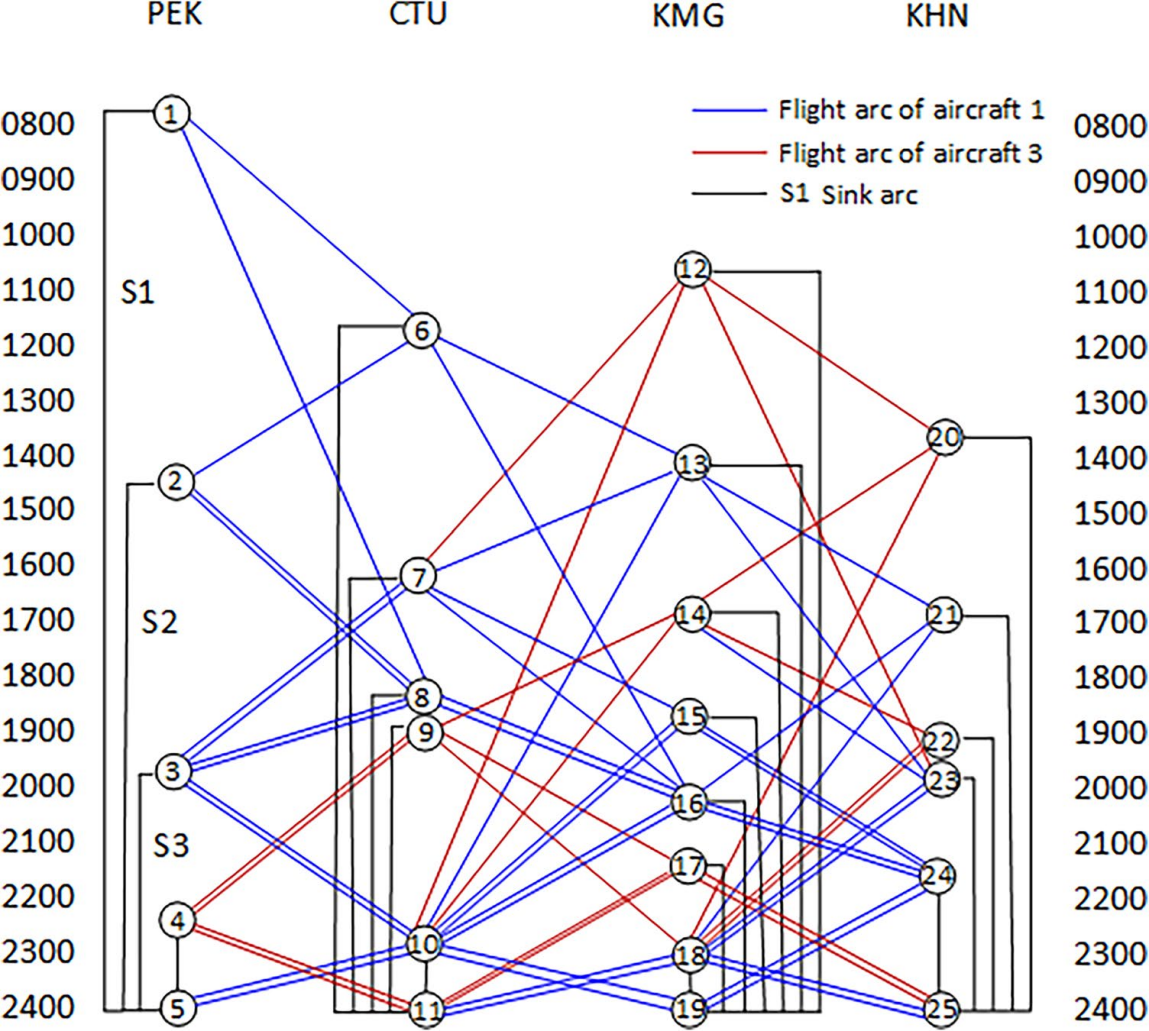
Time-band transformation network for aircraft groundings.

Figure 2 shows the time-band network structure of aircraft fault recovery flights, which is composed of network nodes and flight arcs. The network node represents a specific period, and the flight arc pointing to the node represents the arrival of planned flights during that period. The flight arc of the source node indicates that the available aircraft of that node will fly to the next destination airport. After the flight schedule is converted into a time-band network, it generally includes three types of network nodes: Source node (such as Node 1), intermediate node (such as Node 6), and sink node (such as Node 5); Two Type flight arcs: flight arcs (such as 3U11), replication arcs (such as arcs between nodes 2–8), and sinking arcs (such as S1, S2, S3).

Due to the malfunction of Aircraft 2, only Aircraft 1 and Aircraft 3 are available in the available aircraft set. The flights designated for Aircraft 2 will be executed by Aircraft 1 instead. Figure 2 shows all possible scenarios after the flights have been adjusted, including 25 nodes and 87 arcs, among which Node 5, Node 11, Node 19, and Node 25 are sink nodes. Out of 87 arcs, there are 66 flight arcs and 21 sink arcs. The parameters of these nodes and arcs can reflect the time-band mode of each flight. At the same time, in the process of constructing the network, it is possible to fully consider the constraints that flight scheduling should meet, and according to a certain strategy, the delay duration after flight adjustment can be calculated. Then, a new flight plan can be formulated by comprehensively considering the delay costs and cancellation costs.

The delay cost for each flight arc in Fig. 2 was calculated, and the results are listed in Table 2.

- *Scenario 2* The airport is closed for some reason: Assumed that the Chengdu Shuangliu Airport is closed from 16:00 to 19:00 due to the weather.

**Table 2:** Flight arc delay costs of the sample problem.

Flight ID	Origin node	Destination node	Delay cost ($20/min)	Flight ID	Origin node	Destination node	Delay cost ($20/min)
3U11	2	8	8000	3U22	3	10	6000
3U11	3	10	14,000	3U22	4	11	9400
3U11	4	11	17,400	3U23	9	18	600
3U12	7	15	5400	3U23	10	19	6000
3U12	8	16	8000	3U24	17	11	600
3U12	9	17	8800	3U24	18	11	2700
3U12	10	19	14,000	3U31	13	21	3900
3U13	14	9	3400	3U31	14	22	7300
3U13	15	10	5400	3U31	15	24	9300
3U13	16	10	6000	3U31	16	24	11,100
3U13	17	11	8800	3U31	17	25	12,700
3U13	18	11	10,700	3U31	18	25	14,600
3U14	8	3	2600	3U32	21	16	3900
3U14	9	4	3400	3U32	22	18	6500
3U14	10	5	8600	3U32	23	18	7300
3U21	6	2	600	3U32	24	19	12,900
3U21	7	3	6000	3U33	15	24	2000
3U21	8	3	8600	3U33	16	24	3800
3U21	9	4	9400	3U33	17	25	5400
3U21	10	5	14,600	3U33	18	25	7300
3U22	2	8	600	3U34	24	19	2000

**Figure 3:**
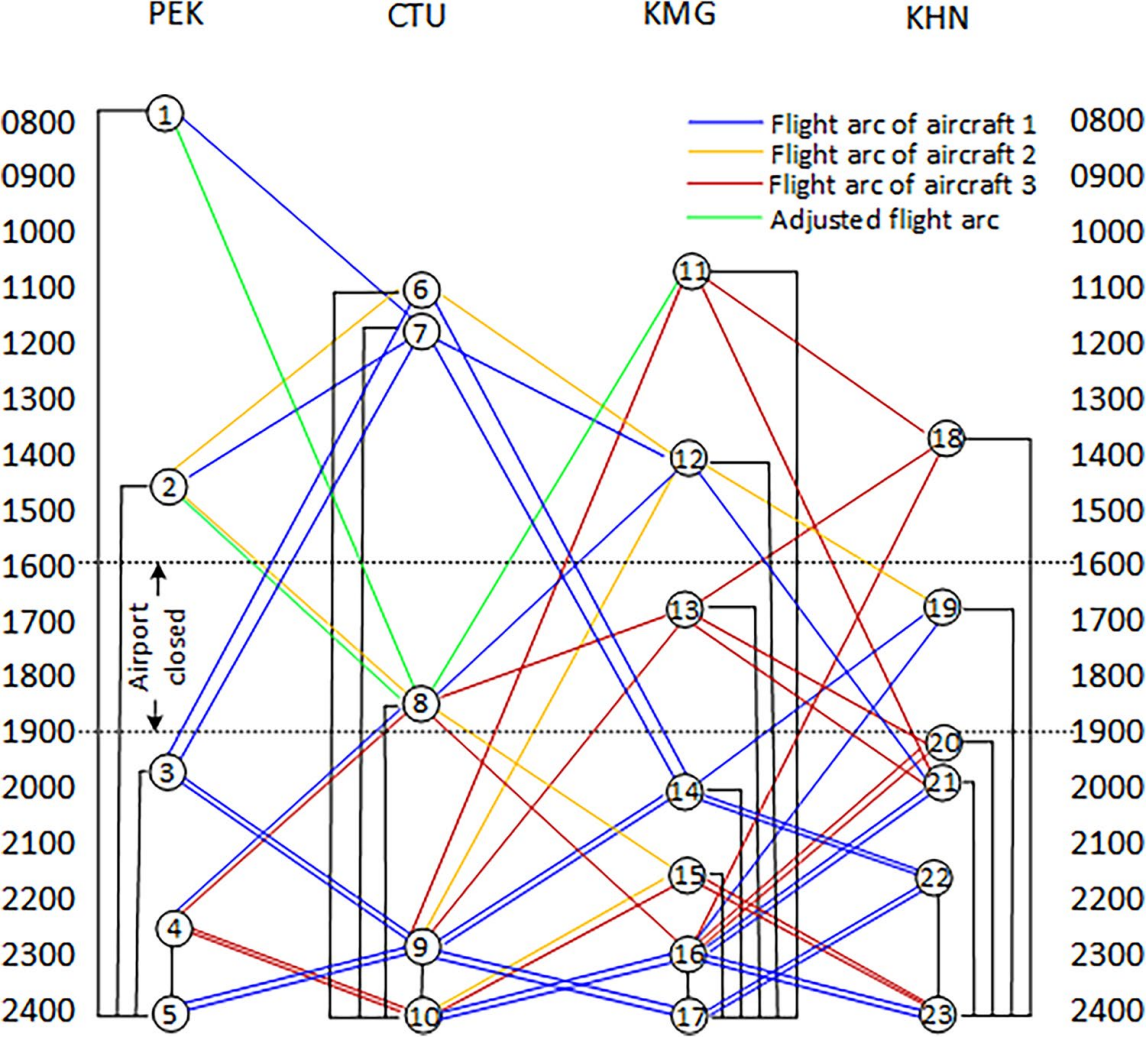
Time-band transformation network for the airport closed.

Figure 3 illustrates the time-band network diagram of airport closure and recovery of flights. Due to the airport being closed for some reason during this period, the aircraft cannot land or take off. Therefore, adjustments need to be made to the originally planned taking off and landing flights during this period. Figure 3 shows the nodes and arcs adjusted for airport closure, consisting of 23 nodes and 72 arcs, respectively. Due to the complexity of the diagram, not all arcs have shown up.

## Binary tree data structure and algorithms

### Binary tree algorithms

The binary tree algorithm is one of those that has been widely applied in computer science. The binary tree seed algorithm is believed to be superior to comparison algorithms in almost all cases^20^. Binary tree and spatiotemporal tunnel method for coarse video time segmentation, which was found to have significantly better computational complexity than the comparison method^21^. The binary tree algorithm can prevent premature and enhance global optimization^22^, reducing the overall delay classification cost^23^. The visibility binary tree algorithm enables robots to run on the shortest path visibility binary tree^24^. Based on the Voronoi diagram, the binary tree algorithm is believed to have tactical significance in anti-ship multi-missile path planning^25^.

Aircraft may experience grounding and delays for any kind of reason during operation. Whenever such events occur, the original flight schedule will suffer. The airline will consequently have to consider assigning alternative flight schedules, reselecting routes for instance, to restore irregular flights at the lowest cost in the shortest possible time. Based on this, this research is supposed to develop an optimal binary tree generation algorithm to cope with this problem.

### Binary tree data structure

**Figure 4:**
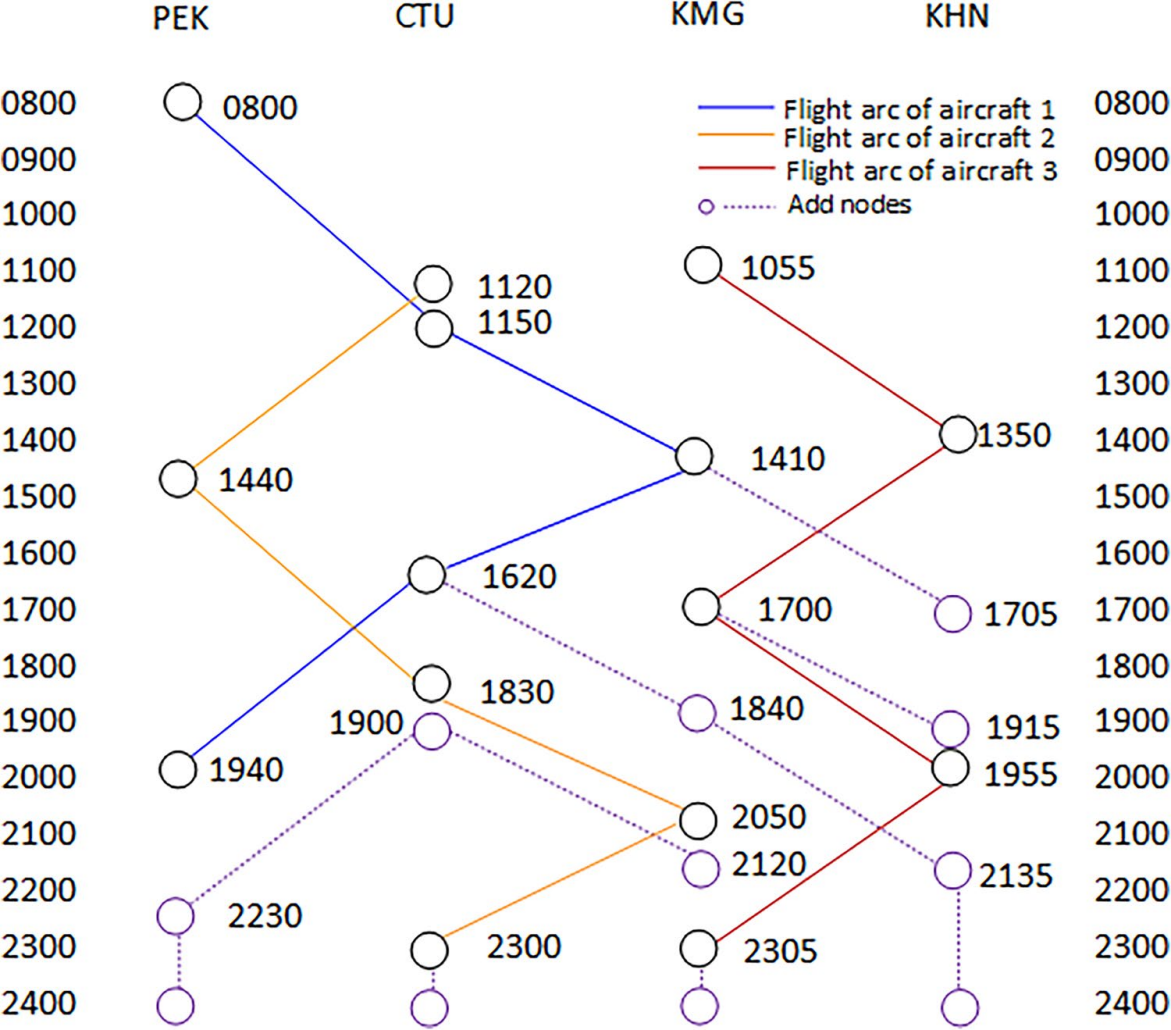
Binary tree data structure.

According to the binary tree principle, a binary tree data structure is constructed, as shown in Fig. 4. The node data in the figure is obtained from Table 1, and the add-node data is calculated based on the aircraft's flight duration and connecting time.

### Mathematical model and algorithms

The use of this binary tree generation algorithm is based on the fact that the solution of the proposed mathematical model is mainly determined by two variables: delay cost and cancellation cost, it is always expected to search and find the minimum cost from potential solutions as it's one of the important operation preferences of Airlines. Second, almost all the restrictions are time-related, including the departure and arrival times of aircraft, airport curfew time, duration of flight, etc. The binary tree generation algorithm is a traversal algorithm, which can search and sort large amounts of data, especially when applied to dense and complex route networks with large amounts of data. Therefore, this algorithm is suitable for solving the mathematical model in this study. This work simulates by programming the binary tree generation algorithm in C++ using Visual Studio 2022.

The definition of parameters and settings of the model are as follows:

- Indices
  *i* = aircraft index; *j* = flight index; *d* = delayed flight ID; *c* = canceled flight ID.
- Parameters
  Airport ID = available airport, allowing aircraft to take off, land, and hold for service; Flight connecting time = shortest connecting time; Aircraft No. = the identification of available aircraft; Flight ID = the number of flight; Number of passengers = calculated based on the number of aircraft seats; Ticket price = calculated based on the total price of passengers’ tickets.
- Variables
  $C_{d} (t)$ = delay costs; $C_{c} (t)$ = cancellation costs.

The mathematical formulations are as follows:

1 $$ F_{min} = min\mathop \sum \limits_{i = 1}^{N} \left[ {C_{d} \left( {t_{j} } \right) + C_{c} \left( {t_{j} } \right)} \right] $$

2 $$ C_{d} (t) = t * p $$

3 $$ C_{c} (t) = n * m $$

*N* = affected flights; *t* = delay time; *p* = delay cost per minute; *n* = number of passengers; *m* = average price.

The description of the constraints is as follows:

- Curfew time: The daily curfew time at the airport is fixed. During the curfew period, aircraft is prohibited from taking off and landing;
- Airport closure: Aircraft cannot take off or land during airport closures;
- Aircraft taking off and landing: The route of each aircraft must be continuous in time and space, must take off from the previous airport, and arrive at the destination airport within the specified flight duration;
- Start and end times of flight: Each flight must depart at the start time and arrive at the destination airport at the arrival time, otherwise there may cause delays;
- Minimum connecting time: The minimum connecting time must be observed between the arrival time of each flight and the subsequent departure time. During the connecting time, aircraft may have ground services such as maintenance, refueling, and catering, etc.;
- The balance constraint of aircraft flow: It’s settled to enforce the utilization of aircraft and ensure the conservation of aircraft flow, maintain the connection between the adjusted flight schedule and subsequent unaffected flight schedules. After completing the last flight, each aircraft must arrive at the sunken node airport to execute the flight schedule the next day.

**Figure 5:**
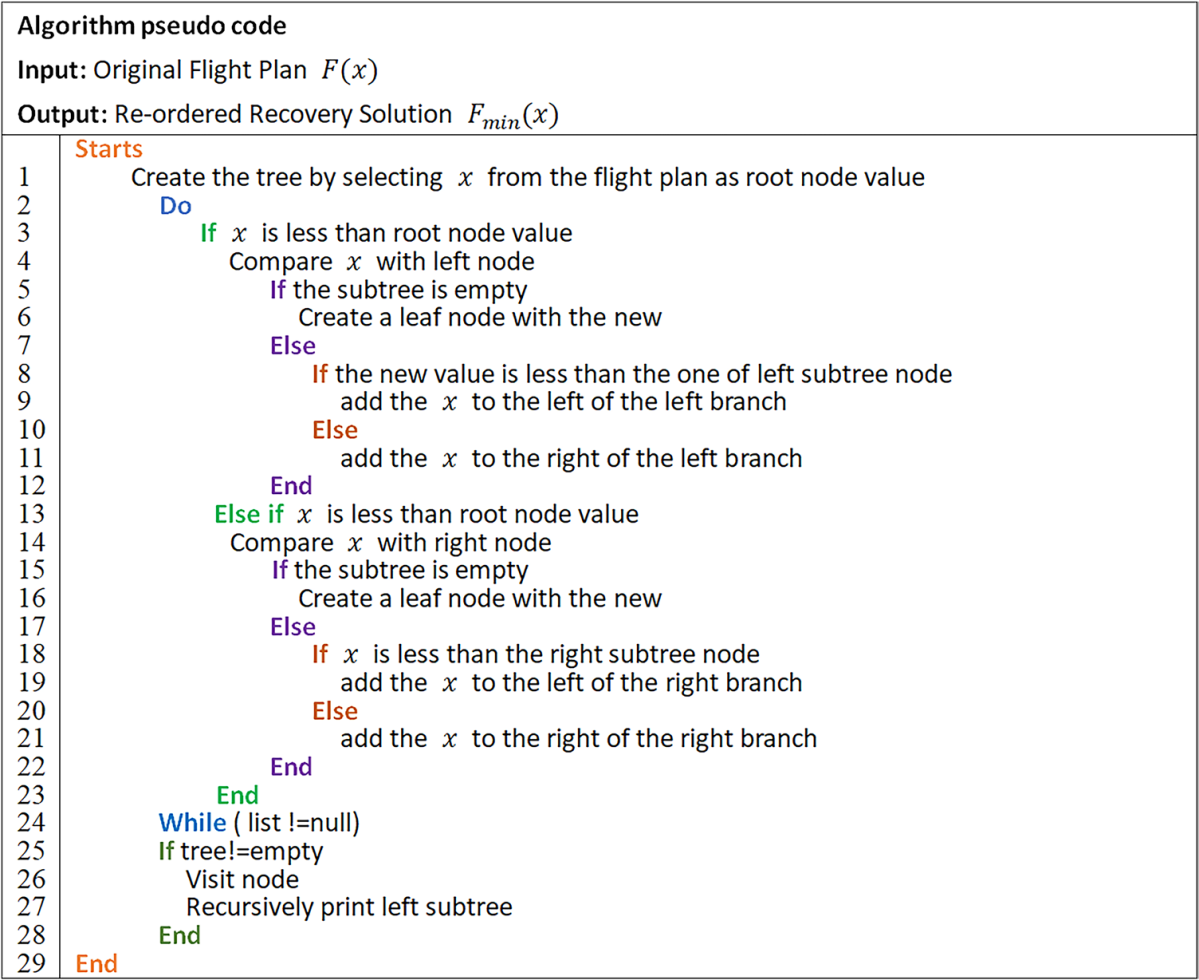
Pseudo-code of binary tree generation algorithm.

The following is the pseudo-code of the algorithm and a brief description of the key steps for solving the model (see Fig. 5).

## Results and discussions

The following are the assumptions used in calculation of this work:

1. The cancellation costs of the flights shall be calculated based on the total ticket price of the passengers on that flight;
2. The delay cost of the flight is calculated at $20 per minute^1^.
3. The connecting time for each flight is 40 min.
4. The operation data used in this study was obtained from Sichuan Airlines and Chengdu Shuangliu Airport in China. The flight information is listed in Table 1. The description of some functions of the program is as follows: The length of delay is determined based on flight status; alternative plans for delay are created for each flight. Binary numbers are used to indicate the status of the flight, with ‘0’ representing flight canceled and ‘1’ indicating flight delayed.

- The results of the **Scenario 1**

Assumed that Aircraft 2 was grounded due to a malfunction before takeoff, resulting in the cancellation of four flights, with a total cost loss of $221,483 and a loss rate of 37.97%, by conducting this optimization method, it was determined that only 2 flights were cancelled, the remaining 2 flights were operated by Aircraft 1, and 5 flights were delayed. Due to the original flight schedule being disrupted, it is necessary to calculate the cost of restoring abnormal flights and the flight schedule. The results are shown in Table 3.

**Table 3:** Flight recovery scheme for the Scenario 1 (total cost = $99,430).

Aircraft No	Flight ID	Origin	Destination	Departure	Arrival	Delay cost	Cancellation cost
Aircraft 1	3U11	PEK	CTU	08:00	11:10	–	–
	3U21	CTU	PEK	11:50	14:30	600	–
	3U22	PEK	CTU	15:10	18:20	600	–
	3U23	CTU	KMG	19:00	20:40	600	–
	3U24	KMG	CTU	21:20	22:50	600	–
	3U14	CTU	PEK	23:30	02:10	8600	–
Canceled	3U12	CTU	KMG	–	–	–	42,168
	3U13	KMG	CTU	–	–	–	46,262
Aircraft 3	3U31	KMG	KNH	10:55	13:10	–	–
	3U32	KNH	KMG	13:50	16:20	–	–
	3U33	KMG	KNH	17:00	19:15	–	–
	3U34	KNH	KMG	19:55	22:25	–	–
Total						11,000	88,430

Table 3 shows the calculation results of the costs of restoring irregular flights caused by aircraft malfunctions. The program operation time is 0.05 s. Two flights were cancelled, five flights were delayed, and the total delay time was 550 min. The delay cost is $11,000, and the cancellation cost is $88,430, totaling $99,430. The total cost loss rate has significantly decreased from 37.97 to 20.92%, recovering 17.05% of the total cost loss. The optimal scheme for flight recovery was obtained, and the flight schedule was restored and the aircraft routes were reassigned, as shown in Fig. 5.

**Figure 6:**
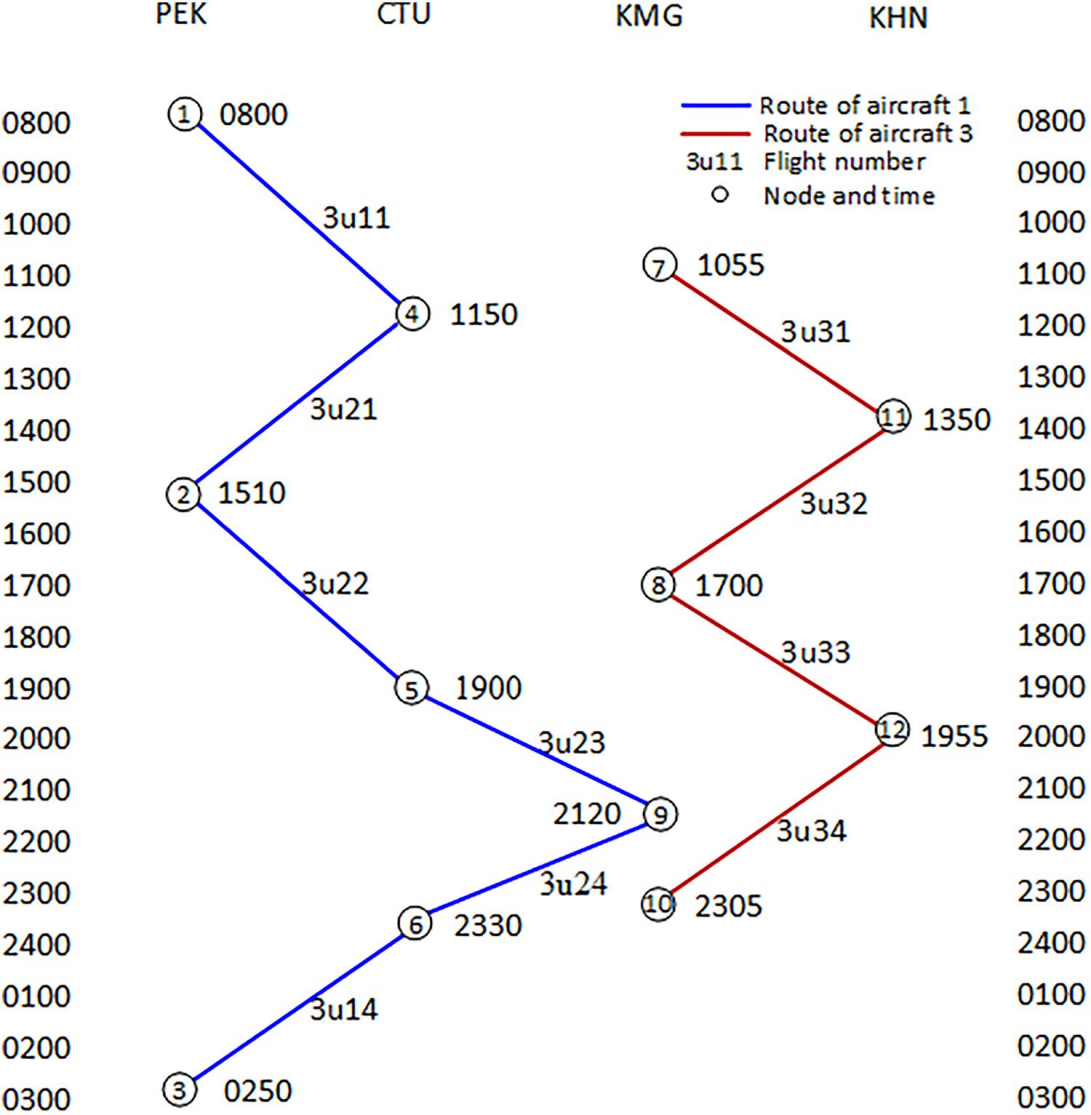
Reassign aircraft routings for the Scenario 1.

Figure 6 shows the reassigned routes after the restoration of irregular flights. Aircraft 1 operates 6 flights on the reassigned routes, while Aircraft 3 operates 4 flights on the original routes. From Fig. 4, it can be seen that Aircraft 1 departed from Beijing Capital Airport at node 1 at 08:00 for flight 3U11 and landed at Chengdu Shuangliu Airport at node 4 at 11:10. 40 min later, flight 3U21 took off from Node 4 at 11:50 and landed at Node 2 at 14:30. Flight 3U22 left Node 2 at 15:10 and arrived at Node 5 at 18:20. Flight 3U23 departed from Node 5 at 19:00 and landed at Node 9 at 20:40. Flight 3U24 took off from Node 9 at 21:20 and arrived at Node 6 at 22:50. In 40 min, flight 3U14 flew from Node 6 at 23:30 and landed at Node 3 at 02:10 the next day. At this point, Aircraft 1 has completed all flights and will stay overnight in Beijing. It can be clearly seen that the reassigned routes for Aircraft 1 are reasonable, with the duration of each flight remaining unchanged and the connecting time meeting the constraint of 40 min, which to some extent has ensured the flight safety and essential order of operation. The results show that the restored flight scheme and the reassigned aircraft routes are valid, while also confirming that the model and algorithm used for solving the problem are reasonable and reliable.

- The results of the** Scenario 2**

Assumed Chengdu Shuangliu Airport was closed due to unforeseen circumstances. During the shutdown period, the flight routes of Aircraft 1 and Aircraft 2 were temporarily interrupted, unable to arrive at the target airport on time, resulting in delays in four flights. Since Aircraft 1 can only stay at Kunming Airport, 3U14 is consequently delayed. Aircraft 2 is staying at Beijing Capital Airport, with delays in 3U22, 3U23, and 3U24. The total delay time in this case is 720 min, and the total cost loss is $14,400, with a loss rate of 2.47%.

Applying this algorithm to solve the model, the computation time is 0.04s. The optimized total delay is 370 min, with a total cost loss of $7400. The total cost loss rate has significantly decreased from 2.47 to 1.27%, recovering a total cost loss of 1.20%. The results show in Table 4.

**Table 4:** Flight recovery scheme for the Scenario 2 (total cost = $7400).

Aircraft no	Flight ID	Origin	Destination	Departure	Arrival	Delay cost	Loss rate (%)
Aircraft 1	3U11	PEK	CTU	08:00	11:10	–	
	3U12	CTU	KMG	11:50	13:30	–	
	3U13	KMG	CTU	14:10	15:40	–	
	3U14	CTU	PEK	19:00	21:40	3200	
Aircraft 2	3U21	CTU	PEK	11:20	14:00	–	
	3U22	PEK	CTU	15:50	19:00	1400	
	3U23	CTU	KMG	19:40	21:20	1400	
	3U24	KMG	CTU	22:00	23:30	1400	
Aircraft 3	3U31	KMG	KNH	10:55	13:10	–	
	3U32	KNH	KMG	13:50	16:20	–	
	3U33	KMG	KNH	17:00	19:15	–	
	3U34	KNH	KMG	19:55	22:25	–	
Total						7400	1.27

**Figure 7:**
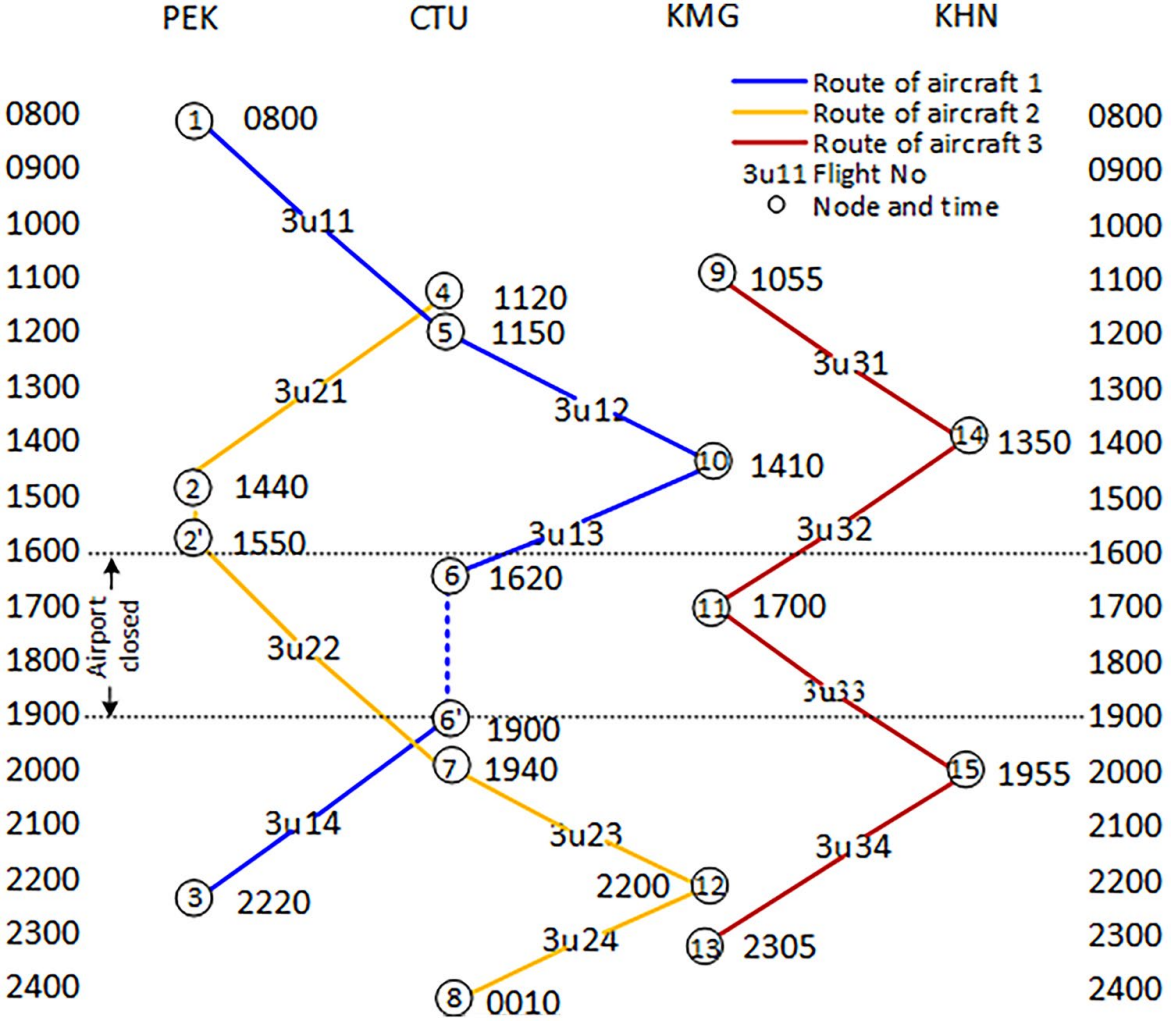
Reassign aircraft routings for the Scenario 2.

Figure 7 shows the reassigned routes after the restoration of irregular flights caused by airport closure. It can be seen that Aircraft 1 had already arrived at Chengdu Shuangliu Airport before the airport was closed. However, due to the airport being closed from 16:00, flight 3U14 can only wait until 19:00 before taking off (see Node 6) and arrive at Beijing Capital Airport at 21:40. Flight 3U22 staying at Beijing Airport can depart at 15:50 during the closure of Chengdu Airport and arrive in Chengdu at 19:00. Afterwards, flight 3U23 will depart from 19:40 and arrive at Kunming Airport at 21:20. 40 min later, flight 3U24 will take off at 22:00 and land at the sink node in Chengdu at 23:30. Aircraft 3 will not suffer from the closure of Chengdu Airport and will fly 4 flights according to the original flight schedule.

From the results, it can be seen that the restored flight arrangements are valid. The rationality and reliability of the model and algorithms proposed in this research have also been further confirmed.

## Conclusions and outlook

The above study suggests that the proposed multimodal time-band network model has distinctive value for figuring irregular flight recovery issues, mainly reflected in the results shown in Figs. 6 and 7. The established mathematical model for restoring irregular flights is reasonable and reliable. This model takes delay cost and cancellation cost as variables, and under constraint conditions, combined with the binary tree generation algorithm developed in this paper, not only achieves the minimization of flight recovery cost and improves spatiotemporal computing efficiency, but also finds from experimental results that this method can help achieve aircraft flow balance, which assists to ensure flight safety. The method proposed in this article will help airlines develop more comprehensive flight plans, enabling resume flight operations in the shortest possible time and at the lowest cost in the event of unexpected situations, thereby improving operational efficiency and profitability.

In addition, the time-band network model and binary tree generation algorithm proposed in this article for restoring irregular flights still have the potential for improvement. In the follow-up work, it can be considered to increase the calculation of the cost of aircraft replacement and improve the binary tree data structure by adding more intermediate nodes. In addition, this study did not consider the influencing factors of the air traffic control department in aircraft flow control. Subsequent research can appropriately obtain this information through relevant systems, further optimize the model, and make the solution to the problem of restoring flights more comprehensive.

## Data Availability

The data collected and used in this paper was obtained from Sichuan Airlines and Chengdu Shuangliu International Airport. The data on flights and the airport information is official and public. For the data and more, please visit the website: https://www.cdairport.com/en/.
